# Sero-Prevalence and Incidence of A/H1N1 2009 Influenza Infection in Scotland in Winter 2009–2010

**DOI:** 10.1371/journal.pone.0020358

**Published:** 2011-06-08

**Authors:** Nigel J. McLeish, Peter Simmonds, Chris Robertson, Ian Handel, Mark McGilchrist, Brajendra K. Singh, Shona Kerr, Margo E. Chase-Topping, Katy Sinka, Mark Bronsvoort, David J. Porteous, William Carman, James McMenamin, Andrew Leigh-Brown, Mark E. J. Woolhouse

**Affiliations:** 1 Centre for Infectious Diseases, Ashworth Laboratories, University of Edinburgh, Edinburgh, United Kingdom; 2 Department of Mathematics and Statistics, University of Strathclyde, Glasgow, United Kingdom; 3 Health Protection Scotland, Glasgow, United Kingdom; 4 Roslin Institute and Royal (Dick) School of Veterinary Studies, University of Edinburgh, Edinburgh, United Kingdom; 5 Health Informatics Centre, University of Dundee, Dundee, United Kingdom; 6 Molecular Medicine Centre, University of Edinburgh, Western General Hospital, Edinburgh, United Kingdom; 7 Centres for Immunity, Infection and Evolution, Ashworth Laboratories, University of Edinburgh, Edinburgh, United Kingdom; 8 West of Scotland Specialist Virology Centre, Gartnavel General Hospital, Glasgow, United Kingdom; 9 Institute of Evolutionary Biology, Ashworth Laboratories, University of Edinburgh, Edinburgh, United Kingdom; Dana-Farber Cancer Institute, United States of America

## Abstract

**Background:**

Sero-prevalence is a valuable indicator of prevalence and incidence of A/H1N1 2009 infection. However, raw sero-prevalence data must be corrected for background levels of cross-reactivity (i.e. imperfect test specificity) and the effects of immunisation programmes.

**Methods and Findings:**

We obtained serum samples from a representative sample of 1563 adults resident in Scotland between late October 2009 and April 2010. Based on a microneutralisation assay, we estimate that 44% (95% confidence intervals (CIs): 40–47%) of the adult population of Scotland were sero-positive for A/H1N1 2009 influenza by 1 March 2010. Correcting for background cross-reactivity and for recorded vaccination rates by time and age group, we estimated that 34% (27–42%) were naturally infected with A/H1N1 2009 by 1 March 2010. The central estimate increases to >40% if we allow for imperfect test sensitivity. Over half of these infections are estimated to have occurred during the study period and the incidence of infection in late October 2009 was estimated at 4.3 new infections per 1000 people per day (1.2 to 7.2), falling close to zero by April 2010. The central estimate increases to over 5.0 per 1000 if we allow for imperfect test specificity. The rate of infection was higher for younger adults than older adults. Raw sero-prevalences were significantly higher in more deprived areas (likelihood ratio trend statistic = 4.92,1 df, P = 0.03) but there was no evidence of any difference in vaccination rates.

**Conclusions:**

We estimate that almost half the adult population of Scotland were sero-positive for A/H1N1 2009 influenza by early 2010 and that the majority of these individuals (except in the oldest age classes) sero-converted as a result of natural infection with A/H1N1 2009. Public health planning should consider the possibility of higher rates of infection with A/H1N1 2009 influenza in more deprived areas.

## Introduction

Accurate knowledge of the fraction of the population infected with influenza and how this changes through time is vital for tracking epidemics and deciding upon intervention strategies [1]. Given widespread under-reporting of cases and largely unknown variability in reporting patterns, sero-positivity (i.e. detection of specific antibodies in serum) is typically the best available measure of population-level infection with influenza [2], [3]. However, estimates of sero-prevalence must be corrected for both a) cross-reactivity in baselines titres resulting from prior infection or immunisation with antigenically similar viruses (which gives the serological test imperfect specificity), and b) the impact of concurrent A/H1N1 2009 vaccination programmes.

In Scotland, the first confirmed case of A/H1N1 2009 was reported on 27 April 2009. Cases continued to be reported until 18 March 2010 and there were a total of 1542 hospitalisations and 69 deaths [4]. However, the estimated total number of cases in Scotland reported to have consulted their GP with flu-like symptoms was just 84,000 (95% confidence interval 79,000 to 92,000) and this is likely to have been a very substantial under-estimate of the true number of cases [5].

Two serological surveys for A/H1N1 2009 influenza have been carried out in the UK during the epidemic, one during the early phase [6] and one during the late phase [7], and there have been several other sero-surveillance studies in other countries worldwide [3], [8]. The one previous report of adult sero-prevalence in Scotland provided only estimates of raw sero-prevalences at a single time point (March 2010), uncorrected for immunisation rates and background cross-reactivity and covering only west Scotland [9]. To address the limitations of that study and to obtain substantive data on the timing of the spread of A/H1N1 2009 and changing incidences of infection during the epidemic, we performed a large-scale sero-surveillance study in the adult population of Scotland during the winter of 2009–2010, covering the latter part of the A/H1N1 2009 epidemic and the immediate post-epidemic period. We were able to correct the raw sero-prevalence data for both background cross-reactivity and the impact of the vaccination programme implemented in Scotland in 2009–2010. This allowed us to estimate the final size of the epidemic (in terms of the fraction infected with A/H1N1 2009) and incidence rates (new infections per 1000 persons per day) during the study period. Using a multivariate approach, the serological data were analysed by age, sex, geographical location and social deprivation to identify risk factors for sero-positivity. Such information is invaluable for assessing the need for and likely impact of interventions to protect public health.

## Methods

### Ethics Statement

Written informed consent was obtained from all participants who took part in the Generation Scotland Scottish Family Health Study. This included consent to: “The information or samples that I provide being used for future medical research into health, illness and medical treatment.” This would have to be approved by a properly constituted research ethics committee. The sero-prevalence project (ref no. GS09033) described here was approved under the Generation Scotland Management, Access and Publication Policy. To satisfy the conditions of the written consent, amendment 19 to 05/S1401/89, describing the use of serum samples for the purposes of project GS09033, was submitted to the Tayside Committee on Medical Research Ethics A. A favourable ethical opinion of the amendment was received dated 30/10/09.

### Samples

Serum samples were provided through the Generation Scotland (GS) Biobank ([10]; see also www.generationscotland.org) from anonymous volunteers in Scotland. A total of 1622 samples were collected from general practices in East Scotland and the greater Glasgow area and were accessed via GS collection centres at Ninewells Hospital in Dundee and Gartnavel Hospital in Glasgow over the periods 22 November 2009 to 18 April 2010 and 20 April 2010 to 28 June 2010 respectively.

### Serology

Detection of A/H1N1 2009-specific antibodies was performed using a microneutralisation (MN) assay based on the HPA protocol [11]. Inactivated human sera were screened at a 1∶40 dilution against the NIBRG122 SO-H1N1 isolate (100 TCID_50_ per well) obtained from the National Institute for Biological Standards and Control and incubated at room temperature for 2 hours followed by the addition of MDCK cells and further incubation at 37°C for 24 hours. Infectivity was determined by staining cells for the IFA nucleoprotein by ELISA using a specific monoclonal antibody, peroxidase-conjugated anti-mouse IgG and substrate development. Plates were read at 450 nm, and optical densities recorded by plate reader.

To estimate background cross-reactivity given previously reported non-zero baseline sero-positivity in subjects uninfected with A/H1N1 2009 influenza [6], we screened samples from the University of Edinburgh clinical specimen archive. These comprised 267 anonymised samples from subjects drawn from the general population (attendees of orthopaedic outpatient departments in Lothian) collected between March and June 2008 from patients ≥21 years old.

We were not able to estimate test sensitivity directly; however, the MN assay applied to A/H1N1 2009 influenza has an estimated sensitivity of at least 83% [12].

### Demographic data

Information provided from study subjects included age, sex, postcode of residence (for subjects with a missing or invalid residential postcode the postcode of the referring GP was used), and date of sample. We also scored subjects according to the Scottish Index of Multiple Deprivation (www.scotland.gov.uk/Topics/Statistics/SIMD) which is derived from the postcode. We also considered additional postcode-derived variables: population density; urban-rural; and ethnicity (as percentage non-Caucasian).

There is a response bias among those who participate in Generation Scotland leading to an under-representation of males, people in younger (<30) and older (>65) age groups and people in the highest deprivation group, as measured by the Scottish Index of Multiple Deprivation. Proportions in of study subject in various demographic subgroups are recorded in Table 1. These biases were com`pensated for in data analysis by weighting samples back to the population estimates obtained from the General Register Office for Scotland (www.gro-scotland.gov.uk/statistics/theme/population/estimates). Weights were constructed separately for sex, age group in 5 year intervals, and deprivation quintiles and combined using raking. All statistical analyses were based upon the weighted sample using the survey package in R [13]. We note that we do not have information on possible response biases with respect to other risk factors (e.g. household size or occupation) that might affect either the likelihood of influenza infection or the likelihood of influenza vaccination in study subjects.

**Table 1:** Demographic characteristics of the study population, compared with available population estimates for the whole of Scotland (see text).

		No. in sample	% in sample	% in Scotland
Age group (years)	18–24	159	10.2	8
	25–34	199	12.7	14
	35–44	267	17.1	15
	45–59	503	32.2	20
	60–64	225	14.4	5
	≥65	210	13.4	15
Sex	Female	932	59.6	52
	Male	631	40.4	48
SIMD quintiles	1 (High)	199	12.7	20
	2	255	16.3	20
	3	287	18.4	20
	4	479	30.7	20
	5 (Low)	343	21.9	20

### Vaccination data

Immunisation by A/H1N1 2009 vaccination began in Scotland on 26 October 2010. Vaccination was targeted at people who were in influenza clinical risk groups, including health care workers and pregnant women. Unlike seasonal influenza vaccine the pandemic vaccine was not available to all patients aged over 65, but only to those in a clinical risk group. About 56% of people aged 65 years and over are in at least one clinical risk group, while for adults aged under 65 years 18% are in a clinical risk group. From late December, vaccination was extended to all individuals aged between 6 months up to 5 years of age.

Vaccination records for individual study subjects were not available. However, population-level data were available. In Scotland, vaccine uptake for individuals grouped by age was recorded by GPs and weekly electronic extracts of data from all practices were obtained. The first extract was received by Health Protection Scotland on 15 November 2009 with 54% of practices reporting and the final extract was available from 1 June 2010 with 95% of practices reporting.

### Statistical analysis

A total of 1622 samples were tested. Of these, 59 were excluded from this analysis because they had a residential postcode from outside Scotland or no valid postcode information was provided. This left a total of 1563 samples (445 from Glasgow and 1118 from East Scotland).

The data were first analysed using logistic multiple regression models (generalised linear models with binomial errors) to identify risk factors for sero-positivity. Two models were fitted. First, data from all samples collected from 1 March 2010 onwards were included in an analysis of post-epidemic patterns (since there were very few new cases or vaccinations after that date). Risk factors examined were age group, sex, region (Glasgow or East Scotland) and deprivation score and the model allowed for clustering of individuals within postcode zones. To this model we added, singly and together, the postcode-level variables population density (as quartiles), urban-rural (as the categories large urban, other urban, small town and rural) and ethnicity (as percent non-Caucasian). Second, data from all samples was analysed with collection date included as a risk factor using a piecewise model with a log trend (fitted separately for each age group) in risk with time prior to a knot point at 1 March 2010 and a constant trend thereafter.

The second set of analyses estimated trends in sero-positivity due to infection with A/H1N1 2009 by incorporating corrections for background cross-reactivity and vaccination uptake. Here, it was assumed that becoming sero-positive due to immunisation or natural infection were independent and mutually exclusive events. The test result for an individual subject was modelled as:
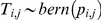


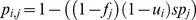
(1)where 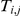 is the serological test result in subject 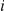 of age group 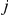, 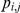 is the probability that subject 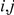 tested positive, 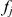 is the probability that subjects in age group 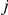 had been infected with A/H1N1 2009, 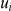 is the probability that patient 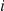 had been vaccinated (estimated from age group-specific final vaccination rates) and became sero-positive and 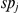 is the serological test diagnostic specificity for age group *j* (which reflects background cross-reactivity).

For the Glasgow samples, because serological testing occurred post-epidemic, 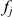 in Equation (1) was assumed to be constant. For the East Scotland samples, which were mainly collected during the epidemic, 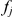 was modelled as increasing to an asymptotic final prevalence as:
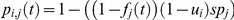


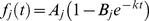
(2)where 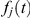 is the probability of that a subject in age group 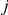 had been infected with A/H1N1 2009 influenza by time 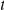, 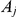 is the asymptotic sero-prevalence in age group 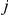, 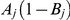 is the sero-prevalence at the start of the study and 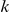 is the negative exponential rate at which sero-prevalence approaches its asymptote (assumed common to all age groups).

Vaccination uptake, 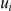, was estimated for each patient by matching their age and sampling date to the relevant regional uptake estimate data (lagged by 14 days to approximate the delay in sero-conversion – see [14]; [5]). Diagnostic test specificity was estimated as described above.

The models were estimated in a Bayesian framework using MCMC simulation. Vague, uniform (0,1) prior distributions were used for 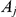 and 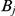 in the East Scotland analysis, and an informative prior distribution, gamma (3,100) was used for 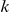. This prior was based on simple empirical analysis of the GP presentation data (data collated from www.hps.scot.nhs.uk/resp/publications.aspx) capturing the mean negative exponential rate of case prevalence approaching its asymptote and giving broad coverage of the variation evident in the different age groups. Sensitivity of the final results to this prior distribution was explored by varying its mean and variance.

The model was realised in JAGS [15] called from the R Statistical System [13]. Parameter estimates on each of 10,000 iterations after a 10,000 iteration ‘burn-in’ period were used to simulate sero-prevalence from infection withA/H1N1 2009 using equations (1) and (2). MCMC chain convergence was assessed by examination of 3 MCMC chains with dispersed initial values and calculation of the Gelman-Rubin statistic [16].

## Results

### Background cross-reactivity

Of the 267 samples collected from subjects in 2008, a total of 9 (3.4%) showed neutralising antibodies to H1N1 using the criteria described in the Methods section. Four of these were from 177 subjects between the ages 21–70 yrs (2.3% sero-positivity), and 5 from subjects >70 yrs old (5.6%). The difference between age groups was not significant (exact P = 0.32). This result is consistent with a low frequency of previous infection or immunisation with antigenically similar H1N1 viruses and corresponds to an estimated test specificity of 96.6% (95% confidence intervals (CIs): 93.9–98.3%). However, because other studies (e.g. [6]) have reported higher background cross-reactivity in older individuals we repeated all analyses using the age-specific estimates.

### Uncorrected sero-prevalence

**Figure 1:**
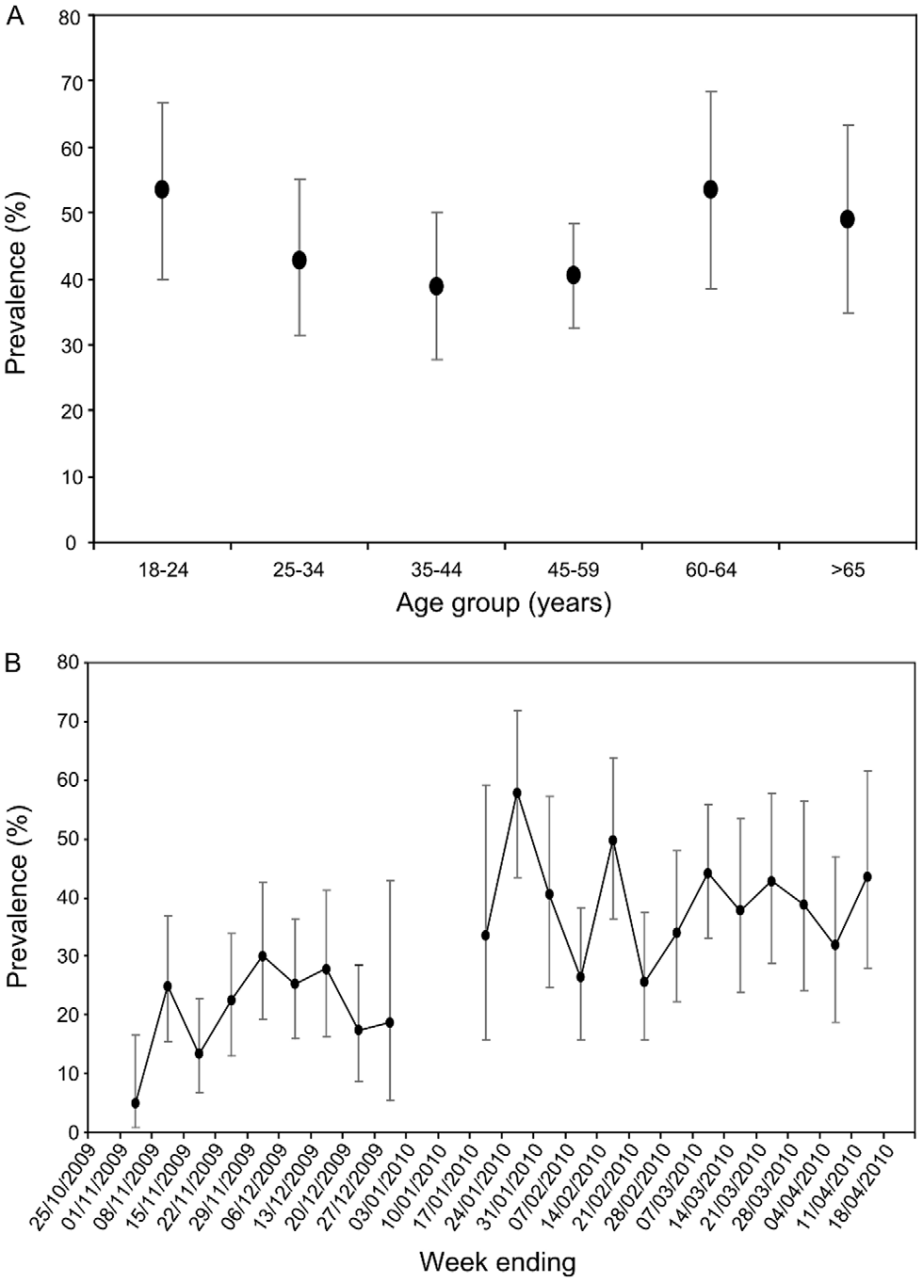
Uncorrected sero-prevalence data. a) Estimates of sero-prevalence by age (in years) in the Glasgow area in spring 2010. Age groups and their corresponding samples sizes are: 18–24 years old, 58; 25–34, 71; 34–44, 76; 45–59, 152; 60–64, 46; >65, 52. Binomial 95% confidence intervals are shown. These results are not corrected for vaccination rates (see Figure 3a). b) Estimates of sero-prevalence by time in East Scotland during winter 2009–2010. Samples sizes range from 16–79 per week (no estimate is given where for sample sizes below 10). Binomial 95% confidence intervals are shown. These results are not corrected for vaccination rates (see Figure 3b).

Of the 1563 samples that met the inclusion criteria, 548 (35%) were sero-positive. Raw sero-prevalences (uncorrected for immunisation, background cross-reactivity or imperfect test sensitivity) at end of epidemic for the Glasgow sample ranged from 40% to 55% between age groups, with lowest sero-prevalences in the 35–44 age category (Fig. 1a). For the East Scotland samples, sero-prevalences substantially increased over the study period (Fig. 1b).

Raw sero-prevalence for all samples collected after 1 March 2010 was 44% (95% CIs: 40–47%). Logistic regression analysis of data from all samples collected after 1 March 2010 did not indicate any effect of age, sex or geographical region on uncorrected sero-prevalence (Table 2). However, there was a significant trend for sero-prevalence to decrease with decreasing deprivation (likelihood ratio trend statistic = 4.92, 1 df, P = 0.03). The odds of an individual in the least deprived areas being sero-positive were 54% of the odds of an individual from the most deprived areas. The deprivation effect was robust to inclusion of population density, urban-rural, ethnicity or all three in the model, and none of these variables were significantly associated with sero-prevalence. Logistic regression analysis of the full data set (allowing for the temporal trends observed in the East Scotland data, Figure 1b) gave very similar results (not shown) with an even stronger relationship with deprivation (likelihood ratio trend statistic = 7.92, 1 df, P = 0.005).

**Table 2:** Odds ratios (OR) from a logistic regression model fitted to data from all post-epidemic samples (collected after 1 March 2010).

		OR	LCL	UCL	P
Age group (years)	18–24	1.000	-	-	
	25–34	0.763	0.421	1.381	0.372
	35–44	0.854	0.477	1.529	0.595
	45–59	0.755	0.454	1.253	0.277
	60–64	0.914	0.488	1.710	0.778
	≥65	1.056	0.531	2.101	0.877
Sex	Female	1.000	-	-	
	Male	0.765	0.541	1.083	0.132
SIMD quintiles	1 (High)	1.000	-	-	
	2	0.638	0.364	1.119	0.118
	3	0.928	0.516	1.670	0.803
	4	0.520	0.292	0.928	0.027
	5 (Low)	0.536	0.304	0.946	0.032
Region	East Scotland	1.000	-	-	
	Glasgow	1.108	0.776	1.581	0.574

The model assumes the same age, gender and deprivation effects in the two regions (interactions with region were all non-significant). LCL and UCL represent lower and upper 95% confidence intervals respectively. SIMD represents the Scottish Index of Multiple Deprivation (see text).

### Vaccination

**Figure 2:**
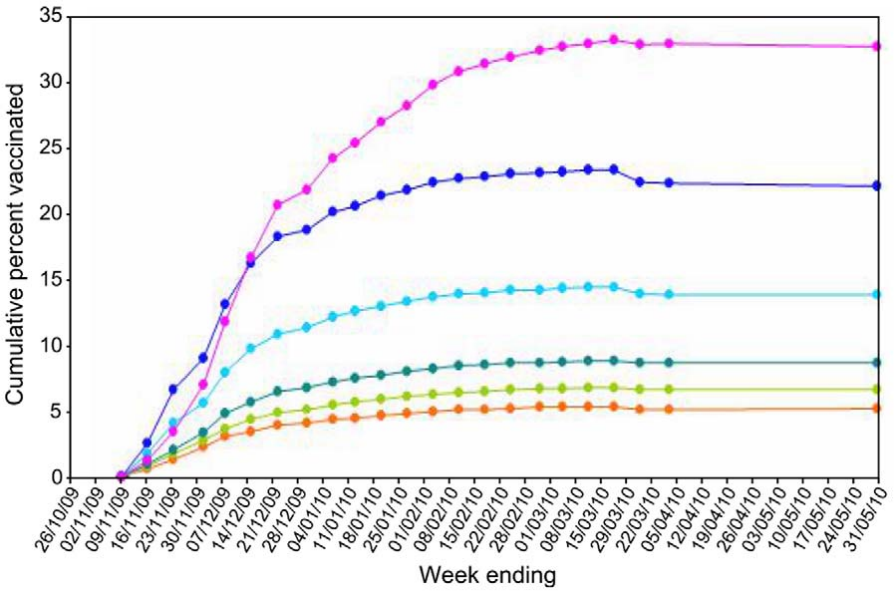
Results from analysis of automated extracts of Scottish national vaccination data provided by Health Protection Scotland (see main text). Cumulative percent general population vaccinated through time is shown by age group: 16–24 yrs old (red line); 25–34 (light green); 35–44 (dark green); 45–59 (turquoise); 60–64 (blue); >65 (pink). Inclusion of additional practices late in the observation period results in a slight (artefactual) dip in the uptake figures.

From the beginning of the vaccination campaign in mid-November 2009 the fraction of the general population vaccinated rose at a decelerating rate until March 2010 (Fig. 2). Final vaccine uptake in the adult population was 15% and increased with age, ranging from <5% in 16–25 year olds to >30% in >65 year olds (Figure 2).

### Corrected sero-prevalence

**Figure 3:**
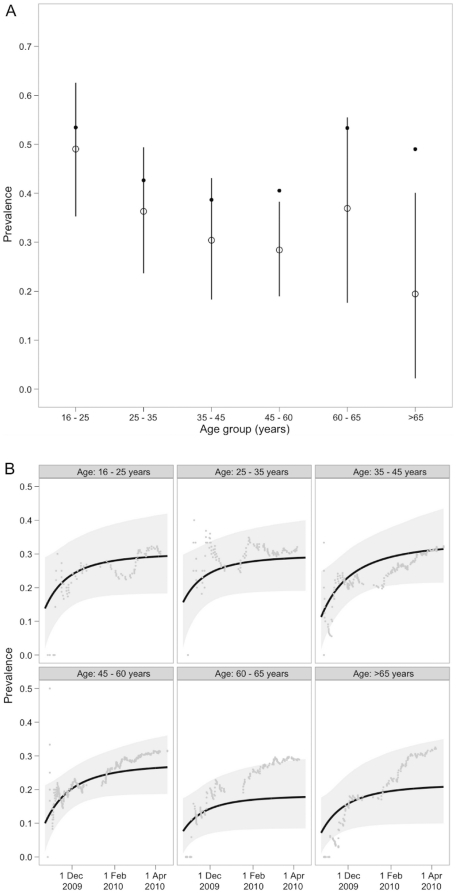
Sero-prevalence estimates corrected for vaccination and background cross-reactivity. a) Estimate of A/H1N1 2009-specific sero-prevalence by age group in the Glasgow sample (mean of posterior distribution shown as open circles and the 95% credible intervals shown as vertical lines). The uncorrected sero-prevalence is shown as black dots. b) Estimate of A/H1N1 2009-specific sero-prevalence for each age group in the East Scotland sample during the study period (mean of posterior distribution is shown as a line with shading showing the 95% credible interval). The uncorrected sero-prevalence is shown over the period as a series of grey dots.

Estimates of age-specific sero-prevalences for the Glasgow sample were corrected for the effects of immunisation and background cross-reactivity (Fig. 3a). Age-standardised, overall sero-prevalence in the adult population was 34% (95% CIs: 27–42%). Central estimates of sero-prevalence by age ranged from 49% in 16–25 year olds to 19% in >65 year olds (but with wide credible intervals). There was a statistically significant decrease in corrected sero-prevalence with age (Spearman's rank correlation for prevalence versus age <0 in >95% model realisations), implying that a greater proportion of uncorrected sero-positivity in older age groups is accounted for by immunisation rather than natural infection (Fig. 2).

Corrected age-specific sero-prevalences in the East Scotland sample varied substantially through time (Fig. 3b). Estimates for the start of the study in late October range from 7% to 16% across age groups (but with wide credible intervals). Across all age groups, approximately 60% infections occurred during the study period, i.e. 40% occurred prior to late October 2009. Corrected sero-prevalences at the end of the epidemic tend to be slightly lower than those estimated for the Glasgow sample, but the confidence intervals overlap for all age groups.

Allowing for a test sensitivity of 83% (see Methods) gave increases in the sero-prevalence estimates of the order of 10%. Allowing for age-specific background cross-reactivity (see Methods) led to only small adjustments in age-specific sero-prevalences (all less than ±2%; well within the credible intervals).

The age-standardised, average estimate of the rate of A/H1N1 2009 infection in the adult population of Scotland at the start of the study period is 4.3 per 1000 persons per day (95% CIs: 1.2 to 7.2) falling close to zero by April 2010. Allowing for age-specific background cross-reactivity led to negligible adjustments to this estimate. However, assuming a test sensitivity of 83% increased estimated initial incidence to 5.6 per 1000 persons per day (95% CIs: 1.6 to 9.4).

## Discussion

The estimated test specificity in this study population was 96.6%, which is consistent with low levels of prior infection with antigenically similar viruses or vaccines in this population. This level of background cross-reactivity is somewhat lower than has been reported elsewhere (e.g. [3], [6], [17], [18], [7]), especially in older individuals. These differences may be due to natural variation in the histories of influenza infection and vaccination in different populations, though differences in test protocols could also play a role.

The sero-prevalence data obtained between late October 2009 and mid-April 2010 from East Scotland indicate a substantial increase (more than two-fold) in cumulative infection with A/H1N1 2009 over the study period, at an initial rate (with some variation across age groups) of at least 4.3 per 1000 persons per day (but with wide confidence intervals), falling almost to zero by April 2010 (Figure 3b). This is one of the first estimates of A/H1N1 2009 incidence based on sero-surveillance data. Baguelin et al. (2011) show the overall rate of change of sero-prevalence for individuals 1–44 years old in England during the second pandemic wave, though that study does not adjust vaccination uptake (which the authors assume to be low).

Our analysis provides an estimate of final size of the 2009–2010 A/H1N1 2009 influenza epidemic (corrected for background cross-reactivity and concurrent immunisation). At least 34% (95% CIs: 27–42%) of the adult population of Scotland are estimated to have been naturally infected with A/H1N1 2009 by 1 March 2010. This is somewhat higher than reported for adult populations in England [5], New Zealand [8] and Pittsburgh PA [18]. Higher adult sero-prevalences reported in England [7] and Norway [19] were not corrected for the effects of vaccination programmes.

In our study there was a trend for corrected sero-prevalence to decrease with age but there was no evidence of differences between sexes or between the two regions of Scotland included in the study. Our data are limited to adults: in children, especially school-age children, rates of natural infection are likely to have been considerably higher [8], [18], [19]. Importantly, our results do suggest that rates of infection with A/H1N1 2009 were higher in more deprived areas, based on the observations that 1) sero-prevalence was significantly higher in more deprived areas but 2) there is no evidence of any relationship between deprivation and immunisation rates [20]. This effect it is not explained by differences in local population density or between urban and rural areas or by differences in ethnicity. No relationship between sero-prevalence and socioeconomic status was found in a study in Australia [21].

Sero-prevalence overall (resulting from a combination of natural infection with influenza A/H1N1 2009, the effects of immunisation programme instigated in late 2009 and low background levels of cross-reactivity) in the adult population of Scotland is estimated to have been at least 44% (95% CIs: 40–47%) by early 2010. This would be expected to generate a significant degree of herd immunity and so, in the absence of significant change in the antigenicity of the virus, protect the population from a major epidemic of A/H1N1 2009 in the 2010–2011 influenza season. However, any such effect would be reduced if: 1) the cut-off antibody titre (1 in 40 dilution) used does not necessarily imply immunity in adults: 2) antibody level and hence protection in vaccinated adults has waned faster than anticipated; 3) relaxation of the pandemic control programme has led to higher transmission rates than in 2009–2010. Further work is needed to examine these possibilities.
